# An evaluation of the Charlson co-morbidity score for predicting sepsis after elective major surgery

**DOI:** 10.4103/0972-5229.78221

**Published:** 2011

**Authors:** Peter A. Hampshire, Arpan Guha, Ann Strong, Dawn Parsons, Patricia Rowan

**Affiliations:** **From:** Department of Critical Care Medicine, Royal Liverpool University & Broadgreen Hospitals NHS Trust, Prescot Street, Liverpool L7 8XP, UK

**Keywords:** Charlson score, predictors, sepsis

## Abstract

**Background and Aims:**

Severe sepsis is a significant cause of morbidity and mortality following major surgery. The Charlson co-morbidity score (CCS) has been shown to be associated with severe sepsis following major surgery for cancer. This prospective observational study investigated the effect of patient factors (CCS, gender, age and malignancy) and intraoperative factors (duration of surgery and allogeneic blood transfusion) on the incidence of sepsis after elective major surgery, and the impact of patient co-morbidities on length of stay in critical care.

**Materials and Methods:**

We prospectively identified a cohort of 101 patients undergoing elective major surgery in a university teaching hospital. The CCS was calculated before surgery, and the incidence of sepsis was documented following surgery. We investigated whether age, malignancy, intraoperative allogeneic blood transfusion, length of surgery or gender were associated with sepsis following surgery.

**Results:**

Twenty-seven (27%) patients developed sepsis. Using multivariate logistic regression, the duration of surgery was associated with the development of sepsis after surgery (*P* = 0.054, odds ratio 1.2). The CCS was not associated with sepsis in this population of cancer and non-cancer patients undergoing elective major surgery, but was associated with longer length of stay in the intensive care unit (*P* = 0.016).

**Conclusions:**

Duration of surgery, but not patient co-morbidity as assessed by the CCS, may predict the postoperative incidence of sepsis. CCS could be used as a guide to predict consumption of critical care resources by elective surgical patients. A higher CCS was associated with a longer ICU stay. Resources, such as postoperative goal directed therapy, may be useful in reducing length of stay, hospital costs and risks of infective complications in this subgroup of patients with higher CCS.

## Introduction

Septic shock and severe sepsis are the major causes of mortality after surgery. The risk of developing sepsis after major surgery increases with emergency operations,[1] the degree of surgical insult,[1] transfusion of allogeneic blood,[2–4] increasing age[5] and male gender.[6,7] The extent of co-morbid illness also increases the risk of postoperative complications, including sepsis, after elective surgery.[1,5,8] The Charlson co-morbidity score (CCS), developed in 1987, is one instrument used to measure the burden of co-morbidity.[9] It uses 19 weighted categories related to chronic health to predict the likelihood of 1-year mortality [Table 1]. Patients with higher levels of chronic co-morbidity as assessed using the CCS are at higher risk of developing complications after surgery, including major head and neck, colorectal and major urological operations.[1,10–12] Although the CCS was not originally designed to identify patients at risk of developing sepsis, patients on chronic dialysis with a high CCS have an increased risk of developing a hospital-acquired infection,[13] and a high CCS is associated with surgical site infections after orthopedic surgery.[14] The CCS is correlated with increased mortality in patients with sepsis caused by bloodstream infections with methicillin-resistant *Staphylococcus aureus*^15^ and *Pseudomonas aeruginosa* pneumonia.[16] Severe sepsis after major surgery for cancer is also associated with an increased CCS.[8]

**Table 1:** Co-morbid conditions with their associated weighted scores

Condition	Weighted score
Myocardial infarction	1
Biventricular heart failure	1
Peripheral vascular disease	1
Cerebrovascular disease	1
Dementia	1
Chronic pulmonary disease	1
Connective tissue disease	1
Peptic ulcer disease	1
Mild liver disease	1
Diabetes with no end-organ damage	1
Hemiplegia from any cause	2
Moderate/severe renal disease	2
Diabetes with end-organ damage	2
Any tumor	2
Leukemia	2
Lymphoma	2
Moderate or severe liver disease	3
Metastatic solid tumor	6
AIDS	6

The Charlson co-morbidity score uses 19 weighted categories, primarily defined using ICD-9-CM diagnoses codes to predict the likelihood of 1-year mortality. Each category has an associated weighted score that is based on the adjusted risk of 1-year mortality. The overall co-morbidity score reflects the burden of co-morbidity: the higher the score, the greater the likelihood of 1-year mortality. The Charlson comorbidity score is obtained by summing the scores associated with the conditions present

Patients who develop severe sepsis after major surgery often require long stays in critical care. The identification of patients who have a high risk of sepsis after surgery may enable critical care resources to be targeted at this high-risk population, reducing unplanned admissions and improving cost-effectiveness. If the CCS was found to correlate with an increased risk of sepsis following surgery, then an increased level of postoperative care could potentially be targeted toward this high-risk population.

## Materials and Methods

The study was conducted in a university teaching hospital that provides a regional service for surgical resection of pancreatic malignancies. The local research ethics committee approved the study protocol and waived the requirement to obtain written informed patient consent since no interventions were made to the treatment of any patient during the study. Consecutive patients aged over 16 years, scheduled for elective major surgery as defined by the OPCS-4.3, were eligible for the study.[17] The operations included major vascular surgery (open abdominal aortic aneurysm repair), major intra-abdominal surgery (bowel resection, nephrectomy, cystectomy), and intrathoracic surgery (esophago-gastrectomies). Patients undergoing elective cardiac surgery were not included, as this surgery is not performed at the hospital. Patients having emergency surgery, those aged 16 years or under and patients in whom sepsis was present or suspected prior to surgery were excluded.

During the study period, three trained research nurses prospectively identified and reviewed the case notes of consecutive patients scheduled for elective major surgery. Data were abstracted from the patients’ case notes using a standardized data collection sheet. The CCS was calculated preoperatively using the criteria defined by Charlson [Table 1]. Patients’ gender, age and the presence or absence of documented malignancy before surgery were recorded.

The following data were recorded after the operation: surgery performed, duration of operation, and number of autologous and allogeneic blood units transfused during surgery. Duration of operation was recorded in minutes and was then rounded off to the nearest quarter hour.

The conduct of anesthesia, and postoperative analgesic technique were decided by the responsible anesthetist. All patients were given appropriate intravenous prophylactic antibiotics at the induction of anesthesia.

The patients were followed up for 10 days after surgery. Evidence of the systemic inflammatory response syndrome (SIRS) was sought from the observation charts and laboratory results daily. Parameters that were not measured on that day (e.g. white cell count) were assumed to be in the normal range when scoring for the presence of the SIRS. If the SIRS criteria were met, possible sources of infection were sought from the case notes, laboratory results, radiographs, computed tomography scan results, or other available imaging. No additional clinical investigations were requested by the data collectors or investigators. The source of sepsis was assigned to a specific site based on the following criteria.

“Chest” was recorded as the source of sepsis if there were one or more of purulent sputum, worsening hypoxemia, new infiltrates on chest radiograph, or positive sputum culture.

“Abdomen” was recorded as the source of sepsis if there was an imaging modality demonstrating an intra-abdominal collection, or growth of pathogenic bacteria in any specimen of intra-abdominal drain fluid.

“Urinary tract” was recorded as the site of infection if there was positive growth of a pathogen in a mid-stream specimen or catheter specimen of urine.

If positive blood cultures occurred without a positive imaging modality or positive microbiological culture from another site, the source of sepsis was attributed to the most likely source of primary infection based on the type of organism cultured from blood, type of surgery performed and clinical signs. The SIRS, sepsis and septic shock were defined as per the American College of Chest Physicians/Society of Critical Care Medicine Consensus Conference criteria.[18]

All admissions to critical care areas (level two or three) were documented, including planned admissions for postoperative care. Where patients were re-admitted to critical care, only the first admission was included in the results. The reason for admission along with length of stay was recorded. Length of stay was recorded in hours and then rounded off to the nearest half day for analysis. “Planned” admissions were defined as those admissions for which a critical care bed was requested prior to the administration of anesthesia.

One investigator randomly analyzed 10% of the completed datasheets and case notes to ensure consistency of scoring and accuracy of the collected data. The data were screened for missing or implausible values, and corrected by discussion with the research nurses. All data were anonymized prior to analysis. Data were entered into a Microsoft Excel spreadsheet prior to analysis (Microsoft Corporation, Redmond, WA, USA). Statistical analysis was performed using SPSS version 17 for Windows (SPSS Inc., Chicago, IL, USA). Where data were missing, normal physiological values were assumed if the presence of the SIRS was being established, and zero was substituted for other non-physiological values.

Comparisons between groups of categorical data were performed using the two-tailed Fisher’s exact test. Comparisons between groups of continuous data were performed using the Mann–Whitney U test. Univariate analyses between groups of categorical data were performed using the Fisher’s exact test. *P* values <0.05 were considered to be statistically significant (two-tailed). Variables associated with sepsis using univariate analysis with a *P* value <0.125 were analyzed using multivariate logistic regression, with sepsis as the dependent variable.

## Results

One hundred and one patients were included in the study. Data on blood transfusion during surgery were missing for two patients. SIRS criteria were missing on one or more days for six patients, hence the SIRS was assumed to be absent on these days.

Sixty-eight (67%) of the patients were males. The median age was 66 [IQR 57–72 (range 16–87)] years. Eighty (79%) patients had documented malignancy prior to surgery. Of the patients in the study, 57 (58%) had lower abdominal surgery, 35 (35%) had upper abdominal surgery, six (6%) had urological surgery and two (2%) had vascular surgery. The median duration of surgery was 4 [IQR 2.5–6.75 (range 0.5–10)] hours. Twenty-eight (28%) patients received allogeneic blood intraoperatively [median units transfused 2, IQR 2–3, (range 1–12)]. Median CCS was 2 [IQR 2–3 (range 0–8)].

Fifty-nine (58%) patients developed SIRS postoperatively. Twenty-seven (27%) patients developed sepsis postoperatively, and this included nine patients who developed septic shock. The source of sepsis was the abdomen (15 patients), the chest (9 patients); and the urinary tract (2 patients). Ten patients had positive microbiological cultures and 17 were positive by clinical evidence (images suggestive of infection on chest or abdominal radiology).

Patients who became septic had longer median duration of surgery (6 hours) than those who did not develop sepsis (3.5 hours) (*P* = 0.003), and were more likely to have received allogeneic blood intraoperatively (48% of the patients who became septic received blood, compared to 20% of the patients who did not develop sepsis) (*P* = 0.013) [Table 2]. The type of surgery performed was not significantly associated with sepsis after surgery (data not shown). There was a greater proportion of male patients in the septic group (78%) compared to the non-septic group (64%) but this was not statistically significant (*P* = 0.233). CCS (*P* = 0.304) and age (*P* = 0.908) were not associated with sepsis. The patients who developed septic shock did not have significantly higher CCS (data not shown). In multivariate analysis, neither the duration of surgery nor the blood transfusion were significantly associated with the development of sepsis after surgery, although the duration of surgery, with a *P* value of 0.054, was of borderline statistical significance [Table 3].

**Table 2:** Characteristics of patients with and without sepsis

	Septic patients	Non-septic patients (*n* = 27)	*P* value (*n* = 74)
Charlson co-morbidity score	2 [2–3 (2–8)]	2 [2–3 (0–6)]	0.308
Duration of surgery (hours)	6 [3.5–8.125 (1.5–10)]	3.5 [2.5–5 (0.5–13)]	0.003
Intraoperative allogeneic blood transfusion	13 (48%)	15 (20%)	0.001
Documented malignancy	23 (85%)	57 (77%)	0.423
Age (years)	64 [59.5–69.5 (50–79)]	66 [56.25–73 (16–87)]	0.908
Male gender	21 (78%)	47 (64%)	0.233

Values are shown as median [IQR (range)], or number (proportion). Continuous data were analyzed with the Mann–Whitney U test. Fisher’s exact test was used to analyze categorical data

**Table 3:** Results of multivariate logistic regression analysis

Variable	*P* value	Odds ratio (95% confidence interval)
Duration of operation	0.054	1.2 (0.99–1.44)
Intraoperative allogeneic blood transfusion	0.100	2.4 (0.85–6.77)

All variables with a *P* value < 0.125 on univariate analysis were entered into the logistic regression analysis, with sepsis as the dependent variable. Duration of operation was entered as a continuous variable. Blood transfusion was entered as a dichotomous variable

Thirty-four (34%) patients were admitted to a critical care area for planned postoperative care. There were 20 admissions to the level three facility (9, 9% unplanned admissions) and 37 admissions to level two facilities (12, 12% unplanned admissions). Eleven patients were admitted to a level two facility and later transferred to a level three unit, which are geographically separate in the hospital. None of the patients admitted to critical care after planned surgery received invasive mechanical ventilation. Overall, 12 (12%) patients were admitted to a critical care bed for unplanned reasons in the first 10 days after surgery. Unplanned admissions therefore accounted for 26% of all admissions to critical care. In six patients, sepsis was the reason for admission. Three patients were admitted to a level three bed with respiratory failure; two of these patients received invasive mechanical ventilation and one received non-invasive continuous positive airway pressure [Table 4].

**Table 4:** Use of critical care resources by patients

	Overall	Planned	Unplanned	*P* value
Admissions	46 (46%)	34 (34%)	12 (12%)	
Level two	37 (80%)*	25 (68%)*	12 (32%)	
Level three	20 (43%)*	11 (55%)*	9 (45%)	
Length of stay (days)				
Level two	2 [1–4 (1–9)]	1.5 [1–4 (1–9)]	2.5 [0.5–4 (0.5–9)]	0.121
Level three	2 [1–8 (1–41)]	2 [1–4 (1–9)]	6.5 [2–19 (1–41)]	0.016

Values are shown as median [IQR (range)], or number (proportion). Continuous data were analyzed with the Mann–Whitney U test

* Eleven patients were admitted to a level two facility and later transferred to a level three unit

In total, the patients consumed 136 level three bed-days and 109 level two bed-days. The median length of stay in both level three and level two facilities was 2 days (IQR 1–8 and 1–4, respectively). Level three length of stay was longer after unplanned admission (median 4, range 0.5–41 days) than after planned admissions (median 0, range 0–4 days, *P* = 0.016). Level three length of stay was associated with increasing CCS (*P* = 0.022), but not with increasing age.

## Discussion

In this study of patients undergoing major elective surgery, we found that sepsis after surgery was not associated with the CCS. However, a higher CCS was associated with an increased level three length of stay.

The strengths of this study were that it was conducted prospectively and all eligible patients were included. No changes were made to the anesthetic given for the surgery or the management of the patients after surgery, so the conditions in the study reflect those that exist in the hospital environment. In this hospital, it is standard practice for patients who are undergoing thoracic, upper and lower gastrointestinal major surgery to be offered an epidural. Epidural analgesia may reduce the incidence of SIRS through an effect on the surgical stress response after surgery,[19–20] although the effect may be limited and the clinical significance is unclear.[21] Despite widespread use of epidural analgesia in this study, 59 (58.4%) patients developed the SIRS postoperatively. This is a lower incidence than that reported in post-surgical intensive care unit patients (93%).[22] However, not all of the patients in the current study were cared for in critical care. The incidence of SIRS in the current study is similar to the number of patients developing SIRS (excluding the first postoperative day) in a similar study where no epidural analgesia was provided (46.2%).[8]

The rate of sepsis after surgery reported in different reports varies, and can be as high as 40.2%.[23] A study on the association of postoperative sepsis with the Charlson score reported an incidence of 20%.[8] Our incidence of sepsis (27%) is comparable with these figures.

There are a number of limitations in this study. The study population included 101 patients, but the number who developed sepsis was relatively small, limiting the ability to detect an association between patient factors previously found to be associated with sepsis. However, our study had a similar number of patients compared to a previous study[8] that had reported an association between a high Charlson score and postoperative sepsis. Secondly, the study was conducted in one institution, which is a university teaching hospital and tertiary referral center for hepatobiliary surgery. Caution should be used before applying our results to other hospitals where the case mix and postoperative care may be different. The specialized nature of study site and its highly selected population limits generalizability of the study.

Thirdly, as we did not evaluate white cell count daily unless a full blood count had been requested by the treating surgeons, patients who would have met the SIRS criteria with a high white cell count may have been classified as not having SIRS (false negatives). In turn, this could have resulted in a number of patients with sepsis not being identified as such.

In a previous study by Mokart and colleagues, a high CCS was associated with severe sepsis.[8] In this study, we found no association between CCS and sepsis. Apart from the different endpoints used in the two studies (sepsis in this study, severe sepsis in the former study), there were differences in the patient populations enrolled into the two studies, which may explain this discrepancy. The patients in the current study had a lower average CCS (a median of 2 in this study compared to a mean of 5 in the Mokart study).[8] Other studies looking at the effect of a higher CCS on outcome have found an adverse effect only when the CCS is higher than 5,[15] so it may be that in our subgroup of patients with sepsis, the level of co-morbidity was not high enough to have any effect. Secondly, only patients who had operations longer than 5 hours were included in the Mokart study,[8] whereas we included all patients who were planned to have major elective surgery on an “intention-to-treat” basis, even if the actual surgical time was short. Finally, all patients in the previous study were admitted to a critical care area after surgery. The present study adds to the debate as to whether the Charlson score will always reliably predict the incidence of postoperative sepsis.

We did not find that male gender or age, as reported previously,[5,7,8,23,24] were significantly associated with sepsis. The previously reported difference in the occurrence of sepsis between males and females may be due to hormonal differences in estrogen levels.[25] However, the exact reason is unclear.

It has been reported that blood transfusion during surgery is associated with infections after surgery.[4,8] In this study, we found an association between blood transfusion and sepsis on univariate analysis that became nonsignificant on multivariate analysis [Tables 2 and 3]. This suggests that the apparent association of blood transfusion with postoperative sepsis may be explained by confounding with longer operating times. Blood transfusions during surgery may confound with prolonged, difficult surgery; intraoperative hypotension; urinary catheterization; or tracheal intubation - factors which could also be associated with the development of sepsis after surgery.[3,26]

We found that the length of operation, on multivariate analysis, may be associated with sepsis postoperatively, with a *P* value of 0.054. This suggests that after technically demanding surgery or when complications occur, sepsis is more likely. This is consistent with previous studies that demonstrated an increased risk of wound infection as the duration of surgery increased.[27,28]

Unplanned admissions consumed a greater number of critical care days than planned admissions and the length of stay was significantly longer after unplanned admission (median 6.5 days) than after planned admission (median 2 days, *P* = 0.016). Unplanned admission to intensive care after surgery is associated with a longer hospital length of stay and may be an indicator of patient safety.[6,29] We found that patient co-morbidity, as reflected in a higher CCS, was also associated with a longer level three unit stay, as has previously demonstrated.[30]

It may also be argued that the presence of co-morbidities, such as malignancy, in our study would make patients more prone to developing sepsis after surgery. We agree that this is a distinct possibility. However, there has never been any study that has compared the incidence of postoperative sepsis in cancer with non-cancer patients.[8] Additionally, one would assume that an increased length of stay would also correlate with the presence of co-morbidities. However, this is not inevitable since it has been shown that using a postoperative care pathway in identified patients can significantly reduce length of stay.[31]

In this study, we found that the duration of surgery, but not patient co-morbidity as assessed by the CCS, may predict the incidence of sepsis postoperatively. The CCS could be used as a guide to predict the likely consumption of critical care resources by elective surgical patients, as a higher CCS was associated with a longer level three unit stay in this study.

The targeting of critical care resources using either preoperative optimization[32] or postoperatively using early goal-directed therapy[33] to high-risk surgical populations has been shown to reduce hospital length of stay and improve outcomes. There was a demonstrated decrease in the incidence of infective complications by 40% using preoperative optimization, which consisted of increasing oxygen delivery to 600 ml/min/m^2^ by using intravenous colloid and dopexamine. It has been estimated that in the UK, if about 500 patients annually could be treated with this perioperative protocol,[32] up to 20 lives could be saved. It was also estimated that because of the projected reduction in hospital stay, there should be an annual cost saving of at least £2,000,000 (approx. INR 140,000,000). With increased availability of advanced non-invasive hemodynamic monitoring, this could be carried out outside of a critical care unit, leading to further cost savings.[34]

Additionally, by identifying patients at risk and allocating personnel resources, such as a rapid response Medical Emergency Team (MET), the relative risk of postoperative sepsis can be reduced. The introduction of such a team, which included the duty intensive care doctor and a designated intensive care nurse with an emergency pack with drugs and equipment needed for resuscitation and tracheal intubation, led to a relative risk reduction in postoperative sepsis (74.3%; *P* = 0.0044) as well a reduced hospital length of stay [23.8–19.8 days (*P* = 0.0092)].[35]

## Conclusion

Resources, such as postoperative goal directed therapy and emergency teams for recognized patients at risk, may be useful in reducing length of stay, hospital costs and risks of infective complications. A combination of methods can be used to carry out such identification. We would recommend that these findings be confirmed in larger studies conducted in a number of different hospital types.
